# Advanced Imaging in Methanol Toxicity: Bilateral Putaminal Necrosis and Frontal–Occipital Ischemia—A Case Report

**DOI:** 10.1155/crra/9972530

**Published:** 2026-08-31

**Authors:** Harminder Sandhu, Shabber Syed, Alhassan Alhasson, Zainab Yousif Shukur, Robert Bixler

**Affiliations:** ^1^ Department of Radiology, Corewell Health William Beaumont University Hospital, Royal Oak, Michigan, USA; ^2^ Department of Radiology, Detroit Medical Center, Detroit, Michigan, USA, dmc.org; ^3^ Department of Radiology, School of Medicine, Wayne State University, Detroit, Michigan, USA, wayne.edu; ^4^ Department of Medicine, College of Medicine, University of Kufa, Kufa, Najaf Governorate, Iraq, uokufa.edu.iq

**Keywords:** case report, diffusion-weighted imaging, ischemia, methanol intoxication, putaminal necrosis

## Abstract

Methanol toxicity remains a critical yet challenging diagnosis due to its nonspecific early presentation and potential for delayed intervention. Although methanol itself is nontoxic, its metabolite—formic acid—induces severe metabolic acidosis and neurotoxicity, particularly affecting the basal ganglia and optic nerves. We present a case of a male in his 50s with altered mental status and visual impairment following windshield washer fluid ingestion. Initial computed tomography (CT) was unremarkable, but follow‐up imaging revealed bilateral putaminal necrosis and subcortical white matter involvement, including the frontal and occipital lobes. Diffusion‐weighted imaging (DWI) findings demonstrated acute ischemia and necrosis, underscoring the value of advanced imaging when methanol toxicity is suspected. This case highlights the importance of recognizing characteristic radiological patterns to facilitate timely diagnosis and improve patient outcomes.

## 1. Introduction

Morbidity and mortality from methanol toxicity remain significant in the United States due to challenging diagnoses and delayed care [1]. Methanol, which resembles ethanol, is found in industrial solvents and other products, leading to potential intoxication through a range of exposures including accidental ingestion, intentional self‐harm, recreational consumption, and use within alcohol use disorder patterns [2–4]. Although methanol itself is nontoxic, its metabolite, formic acid, can cause severe toxicity by inhibiting the cytochrome oxidase complex, resulting in histotoxic hypoxia and cell death [5]. Symptoms typically appear 12–24 h postingestion, often starting with visual changes due to optic nerve damage [2]. Severe metabolic acidosis may lead to respiratory failure and death [6, 7]. Treatment includes gastric lavage, ethanol or fomepizole therapy, and hemodialysis, with prognosis dependent on the amount ingested and treatment timing [1]. Early diagnosis via blood gas analysis, serum methanol levels, and radiological imaging is crucial for improving outcomes [2]. Radiologic imaging plays an important role when methanol toxicity is suspected, particularly when the diagnosis is delayed or the initial computed tomography (CT) examination is unrevealing. Magnetic resonance imaging (MRI), especially diffusion‐weighted imaging (DWI), can demonstrate characteristic patterns of injury and detect early ischemic changes that may not be visible on CT [2].

MRI findings in methanol toxicity typically reveal bilateral putaminal necrosis, with or without variable hemorrhage. Hemorrhage is thought to represent a minority of cases, in the range of 25%–33% [8, 9], resulting from the toxic effects of methanol metabolites and metabolic acidosis on the basal ganglia [2, 10]. Formic acid exerts a direct neurotoxic effect, particularly on the putamen [11, 12], which is vulnerable due to its intrinsic microvascular anatomy, high mitochondrial concentration and metabolic demand [7, 11]. Although this finding is characteristic of methanol toxicity, it is not specific and can also occur in conditions such as Wilson, Leigh, and mitochondrial diseases [10]. Severe intoxication can lead to additional neurotoxic effects, including bilateral subcortical white matter necrosis or edema, cerebellar necrosis, diffuse cerebral edema, and optic nerve necrosis [2]. The myelinoclastic effect of formic acid may result in optic neuropathy, causing potential permanent visual impairment [11]. Although bilateral putaminal necrosis is a key imaging finding of methanol toxicity, concomitant diffusion restriction involving both the frontal and occipital subcortical white matter has uncommonly been described, particularly during the acute stage [13–15]. This report presents the imaging findings of a male in his 50s who developed classic radiological findings of methanol toxicity along with necrosis in the frontal and posterior occipital lobes.

## 2. Case

A male in his 50s presented to the emergency room with altered mental status. Due to his condition, he provided limited history, including diffuse body pain and recent ingestion of windshield washer fluid. His status rapidly deteriorated, with impaired ability to follow verbal commands, diaphoresis, mild tachypnea, tachycardia, areflexia, and foaming at the mouth. He became unresponsive and required emergency intubation and ventilation. An orogastric tube drained dark red liquid.

Initial laboratory evaluation showed severe anion gap metabolic acidosis (pH: 6.899, pCO2: 16 mmHg, HCO3: 3.2 mmol/L, anion gap: 24), elevated lactic acid (4.3 mmol/L [< 2]), and a negative drug screen. Nephrology initiated urgent hemodialysis and sodium bicarbonate infusion. The patient received fomepizole, and serum methanol was confirmed at 392 mg/dL.

The patient was extubated on hospital Day 3. On hospital Day 6, he had limited verbal communication, bilateral nonreactive pupils, and endorsed absent vision since admission, gradually improving, consistent with toxic optic neuropathy from methanol intoxication. The patient was then transferred to inpatient rehabilitation and remained hospitalized for 35 days. After which, the patient was discharged home and followed up as an outpatient with physical medicine and rehabilitation. At his last follow‐up, deficits included bilateral decreased vision and reduced right upper extremity active range of motion, strength, and functional use, impacting both basic and instrumental activities of daily living and transfers. Occupational therapy was planned to address right upper extremity deficits, and follow‐up with neuro‐ophthalmology was recommended. Further follow‐up data were not available.

**Figure 1:**
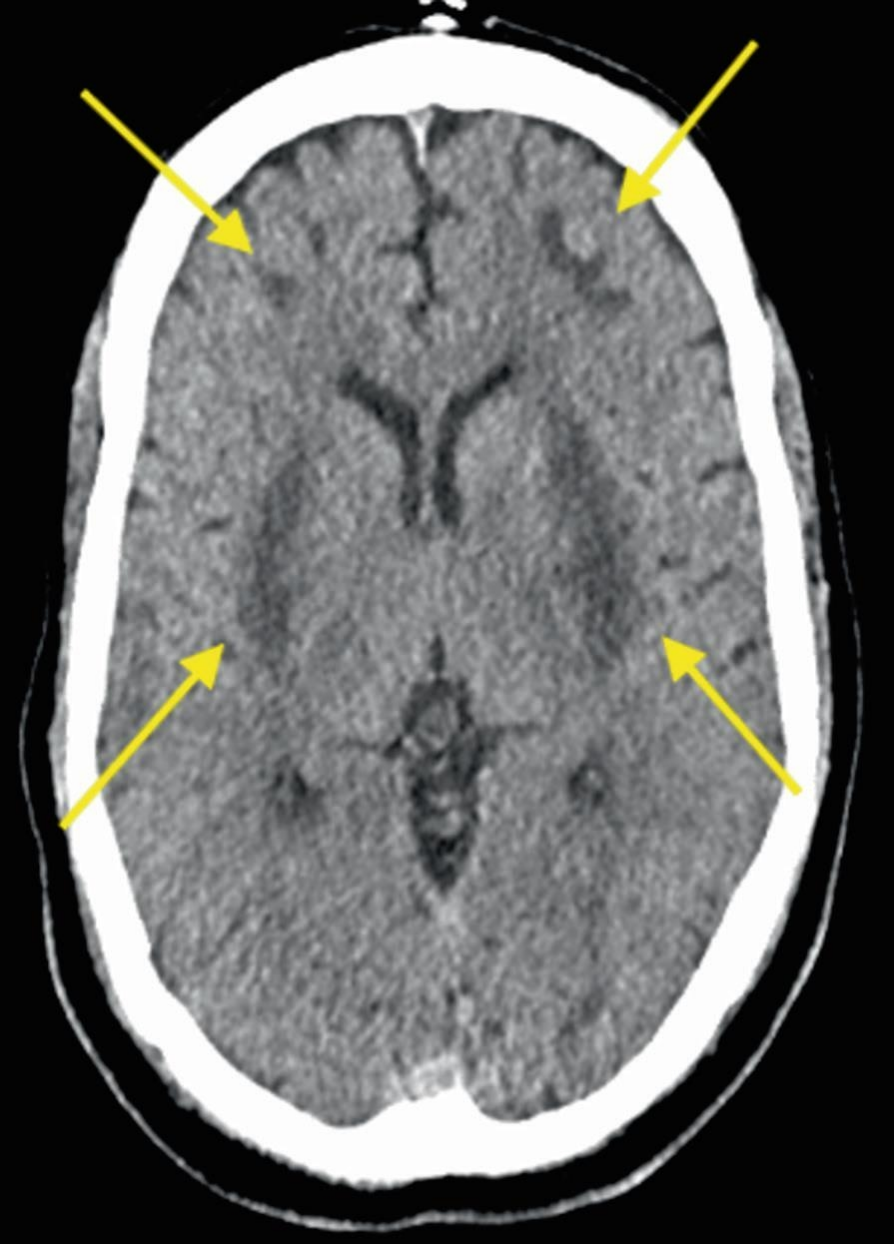
Noncontrast CT Brain (axial view) hospital Day 3 showing diffuse bilateral hypoattenuation of the putamen, external capsule, and claustrum; bilateral frontal lobe subcortical low‐attenuation areas are also shown (yellow arrows). Teaching point: These findings illustrate the evolution of methanol toxicity after an initially normal CT.

**Figure 2:**
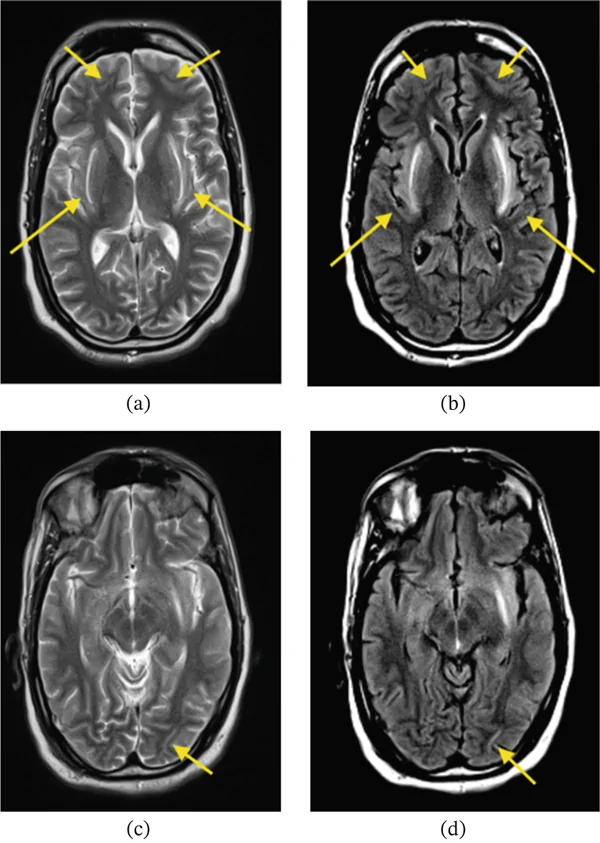
Brain MRI hospital Day 10. (a) T2‐ and (b) FLAIR‐weighted MRI scans at the level of basal ganglia (axial view) showed bilateral putamen, external capsule, and anterior frontal subcortical white matter with abnormal T2/FLAIR hyper‐intensity (yellow arrows). (c) T2‐ and (d) FLAIR‐weighted MRI scans at the level of occipital lobes (axial view) showed left occipital subcortical white matter with abnormal T2/FLAIR hyper‐intensity (yellow arrows). Teaching point: T2/FLAIR sequences demonstrate characteristic bilateral putaminal injury with additional frontal and occipital subcortical white matter involvement.

**Figure 3:**
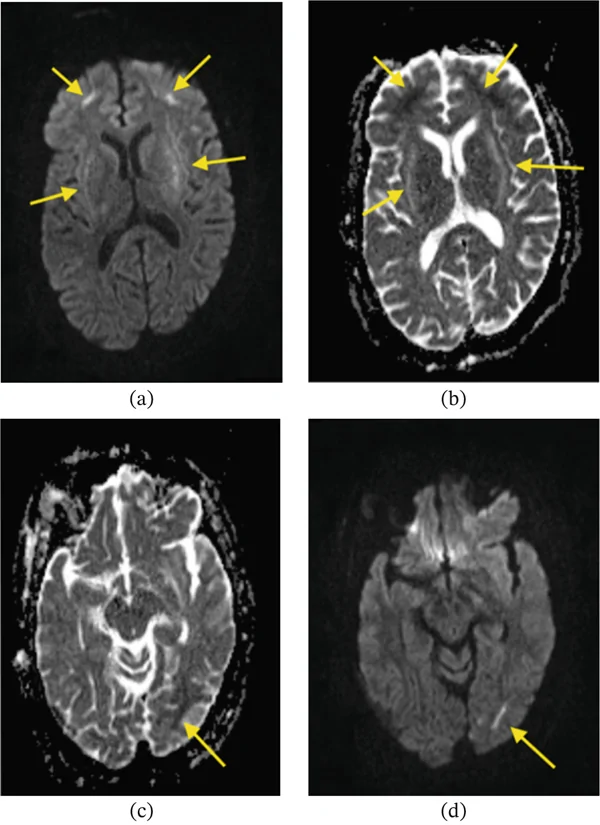
Brain MRI hospital Day 10. (a) DWI and (b) ADC at the level of basal ganglia (axial view) showed bilateral lateral putamen, external capsule, and anterior frontal subcortical white matter with diffusion restriction (yellow arrows). (c) ADC and (d) DWI at the level of occipital lobes (axial view) showed left posterior occipital subcortical white matter with diffusion restriction (yellow arrows). Teaching point: Restricted diffusion confirms acute ischemic injury involving both the basal ganglia and the frontal‐occipital subcortical white matter.

A noncontrast CT of the brain obtained at presentation (hospital Day 0) was unremarkable. On hospital Day 3, a follow‐up CT demonstrated large areas of hypoattenuation involving the bilateral putamina, extending laterally, with external capsule and subcortical insular white matter involvement (Figure 1). On hospital Day 10, T2‐weighted and T2‐fluid‐attenuated inversion recovery (FLAIR) sequences showed hyperintensity in the bilateral putamina, external capsules, and anterior frontal and posterior occipital subcortical white matter (Figure 2). The aforementioned areas also had hyperintense signal on DWI and corresponding hypointense signal on apparent diffusion coefficient (ADC) map, compatible with restricted diffusion (Figure 3).

## 3. Discussion

We presented a classic clinical case of methanol toxicity with characteristic radiological findings. Our patient presented with diffuse body pain and altered mental status, compounded by visual disturbance upon admission. Although the initial CT was unremarkable, a repeat CT showed bilateral hypodense putamina and frontal subcortical white matter involvement. Follow‐up MRI demonstrated cytotoxic edema with hyperintense T2 signal and restricted diffusion in the bilateral putamina and subcortical frontal and occipital white matter, as can be seen with necrosis.

Initial unremarkable findings on CT imaging can occur in methanol toxicity [16]. Classically, bilateral putaminal necrosis becomes apparent on images with sparing of globi pallidi. When visible, the necrosis can be seen on CT as hypodense lesions, whereas on MRI this necrosis is seen as hyperintense T2 signaling [16]. Subcortical white matter, along with cerebellar and optic nerve involvement, can also be appreciated depending on toxicity level [17]. Although hemorrhage was not shown in our case, hemorrhagic necrosis can develop, which can alter the radiologic findings depending on the extent, compartment of hemorrhage, and age of blood products [18].

Several other phenomena also have a predilection for the basal ganglia. Leigh disease and Wilson disease can also manifest as hyperintense T2 signal on MRI in the putamen [18]. Basal ganglia findings on CT and MRI also have a wide differential including carbon monoxide poisoning, vigabatrin use, and uremic encephalopathy. Carbon monoxide poisoning characteristically affects the globi pallidi with rare manifestation in the putamen. Vigabatrin typically affects the globi pallidi as well, whereas the most common pattern of uremic encephalopathy affects the white matter surrounding the basal ganglia commonly seen as the lentiform fork sign [17]. In this patient, initially normal CT findings evolved into characteristic bilateral putaminal necrosis with additional frontal and occipital white matter diffusion restriction on MRI. This case illustrates the value of MRI, particularly DWI, in demonstrating the extent of methanol‐related brain injury.

## 4. Conclusion

This case adds to the limited literature documenting the classical radiologic findings of methanol toxicity across imaging modalities. The patient presented with diffuse pain, altered mental status, and visual disturbance, with an initially unremarkable CT scan. Follow‐up MRI revealed bilateral putaminal necrosis with subcortical white matter involvement, particularly in the frontal and occipital lobes—areas not commonly highlighted in prior reports. DWI findings demonstrated acute ischemia in these regions, offering valuable insights into the broader neurotoxic effects of methanol. Recognizing these imaging patterns alongside clinical symptoms is crucial for timely diagnosis and improved outcomes in methanol toxicity.

## Funding

No funding was received for this manuscript.

## Ethics Statement

Institutional Review Board (IRB) approval was not required for this study because it is a single‐patient case report.

## Consent

The study follows ICMJE guidelines. All patient data has been deidentified with anonymity ensured for patient safety.

## Conflicts of Interest

The authors declare no conflicts of interest.

## Data Availability

Data sharing not applicable to this article as no datasets were generated or analyzed during the current study.

## References

[1] Ghannoum M. , Hoffman R. S. , Mowry J. B. , and Lavergne V. , Trends in Toxic Alcohol Exposures in the United States From 2000 to 2013: A Focus on the Use of Antidotes and Extracorporeal Treatments, Seminars in Dialysis. (2014) 27, no. 4, 395–401, 10.1111/sdi.12237. 24712848

[2] Blanco M. , Casado R. , Vázquez F. , and Pumar J. M. , CT and MR Imaging Findings in Methanol Intoxication, American Journal of Neuroradiology. (2006) 27, no. 2, 452–454, 16484428. 16484428 PMC8148792

[3] Mahdavi S. A. , Zamani N. , McDonald R. , Akhgari M. , Kolahi A. A. , Gheshlaghi F. , Ostadi A. , Dehghan A. , Moshiri M. , Rahbar-Taramsari M. , Delirrad M. , Mohtasham N. , Afzali S. , Ebrahimi S. , Ziaeefar P. , Khosravi N. , Kazemifar A. M. , Ghadirzadeh M. , Farajidana H. , Barghemadi T. , Sadeghi F. , Hadeiy S. K. , Hadipourzadeh M. , Mesbahi J. , Malekpour M. R. , Arabi M. , Jamshidi F. , Dadpour B. , Hovda K. E. , and Hassanian-Moghaddam H. , A Cross-Sectional Multicenter Linkage Study of Hospital Admissions and Mortality Due to Methanol Poisoning in Iranian Adults During the COVID-19 Pandemic, Scientific Reports. (2022) 12, no. 1, 10.1038/s41598-022-14007-1, 35697919. PMC9189800 35697919

[4] Perkins J. E. , Hovda K. E. , Chowdhury F. R. , Sørensen J. B. , Eddleston M. , and Street A. , Alcohol as Poison: A Narrative Review of Social Science Scholarship relevant to Methanol Poisoning in Low- and Middle-Income Countries, Alcohol and Alcoholism. (2025) 60, no. 3, agaf018, 10.1093/alcalc/agaf018, 40294119. 40294119 PMC12036659

[5] Wu X. , Gu M. , Wang W. , Zhang H. , and Tang Z. , Case Report: Early Recognition, Treatment, and Occupational Safety Protection are Crucial for Methanol Toxicity, Frontiers in Medicine. (2022) 9, 10.3389/fmed.2022.918812, 918812. 35774994 PMC9237385

[6] Onder F. , Ilker S. , Kansu T. , Tatar T. , and Kural G. , Acute Blindness and Putaminal Necrosis in Methanol Intoxication, International Ophthalmology. (1998) 22, no. 2, 81–84, 10.1023/a:1006173526927, 10472766. 10472766

[7] Viterbo G. G. , Urquiza S. C. , and Fragante R. E. , Intracranial and Optic Nerve MRI Findings From Methanol Toxicity in a Young Adult Male Patient Presenting With Blurring of Vision, Eurorad. (2021) 23, https://www.eurorad.org/case/17254.

[8] Zakharov S. , Kotikova K. , Vaneckova M. , Seidl Z. , Nurieva O. , Navratil T. , Caganova B. , and Pelclova D. , Acute Methanol Poisoning: Prevalence and Predisposing Factors of Haemorrhagic and Non-Haemorrhagic Brain Lesions, Basic & Clinical Pharmacology & Toxicology. (2016) 119, no. 2, 228–238, 10.1111/bcpt.12559, 26806851. 26806851

[9] Taheri M. S. , Moghaddam H. H. , Moharamzad Y. , Dadgari S. , and Nahvi V. , The Value of Brain CT Findings in Acute Methanol Toxicity, European Journal of Radiology. (2010) 73, no. 2, 211–214, 10.1016/j.ejrad.2008.11.006, 19101105. 19101105

[10] Hsu H. H. , Chen C. Y. , Chen F. H. , Lee C. C. , Chou T. Y. , and Zimmerman R. A. , Optic Atrophy and Cerebral Infarcts Caused by Methanol Intoxication: MRI, Neuroradiology. (1997) 39, no. 3, 192–194, 10.1007/s002340050391, 9106292. 9106292

[11] Gaul H. P. , Wallace C. J. , Auer R. N. , and Fong T. C. , MR Findings in Methanol Intoxication, American Journal of Neuroradiology. (1995) 16, no. 9, 1783–1786, 8693975. 8693975 PMC8338220

[12] Chen J. C. , Schneiderman J. F. , and Wortzman G. , Methanol Poisoning: Bilateral Putaminal and Cerebellar Cortical Lesions on CT and MR, Journal of Computer Assisted Tomography. (1991) 15, no. 3, 522–524, 2026826. 2026826

[13] Camurcuoglu E. and Halefoglu A. M. , CT and MR Imaging Findings in Methanol Intoxication Manifesting with BI Lateral Severe Basal Ganglia and Cerebral Involvement, Journal of the Belgian Society. (2022) 106, no. 1, 10.5334/jbsr.2836, 35859917. PMC9267022 35859917

[14] Ahsan H. , Akbar M. , and Hameed A. , Diffusion Weighted Image (DWI) Findings in Methanol Intoxication, Journal of the Pakistan Medical Association. (2009) 59, no. 5, 321–323, 19438141. 19438141

[15] Elkhamary S. M. , Fahmy D. M. , Galvez-Ruiz A. , Asghar N. , and Bosley T. M. , Spectrum of MRI Findings in 58 Patients With Methanol Intoxication: Long-Term Visual and Neurological Correlation, Egyptian Journal of Radiology and Nuclear Medicine. (2016) 47, no. 3, 1049–1055, 10.1016/j.ejrnm.2016.06.011.

[16] Spampinato M. V. , Castillo M. , Rojas R. , Palacios E. , Frascheri L. , and Descartes F. , Magnetic Resonance Imaging Findings in Substance Abuse: Alcohol and Alcoholism and Syndromes Associated With Alcohol Abuse, Topics in Magnetic Resonance Imaging. (2005) 16, no. 3, 223–230, 10.1097/01.rmr.0000192175.26243.a7. 16340646

[17] de Oliveira A. M. , Paulino M. V. , Vieira A. P. F. , McKinney A. M. , da Rocha A. J. , dos Santos G. T. , Leite C. C. , Godoy L. F. S. , and Lucato L. T. , Imaging Patterns of Toxic and Metabolic Brain Disorders, Radiographics. (2019) 39, no. 6, 1672–1695, 10.1148/rg.2019190016. 31589567

[18] Sharma P. , Eesa M. , and Scott J. N. , Toxic and Acquired Metabolic Encephalopathies: MRI Appearance, American Roentgen Ray Society. (2009) 193, no. 3, 879–886, 10.2214/AJR.08.2257. 19696305

